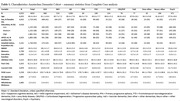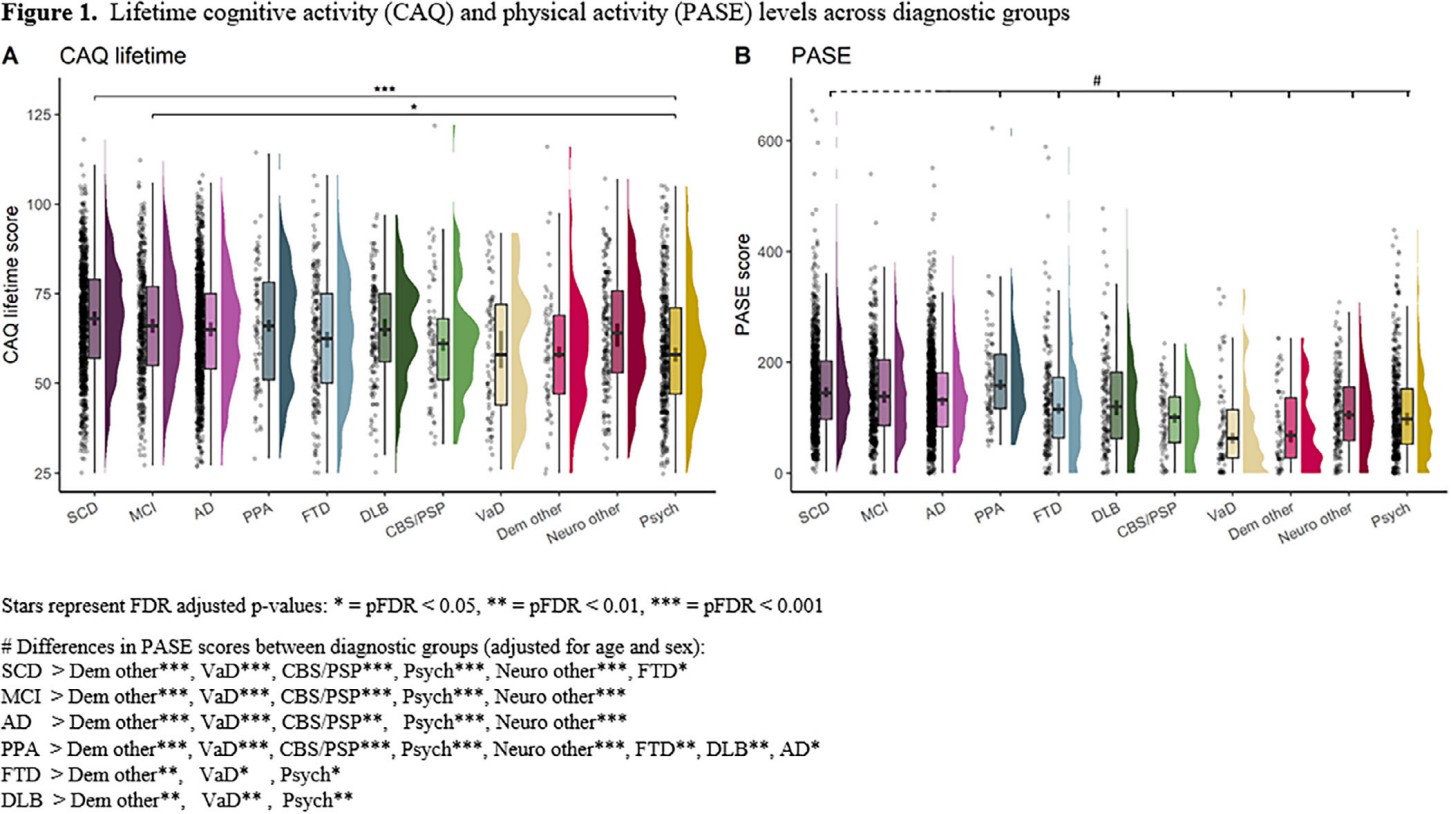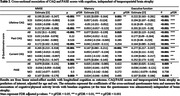# Cognitive and physical activity are related to increased cognitive reserve in a large memory clinic cohort

**DOI:** 10.1002/alz.092443

**Published:** 2025-01-09

**Authors:** Diana I. Bocancea, Anna C. van Loenhoud, Ismael Luis Calandri, Vikram Venkatraghavan, Colin Groot, Casper de Boer, Yolande A.L. Pijnenburg, Frederik Barkhof, Wiesje M. van der Flier, Rik Ossenkoppele

**Affiliations:** ^1^ Alzheimer Center Amsterdam, Department of Neurology, Amsterdam Neuroscience, Vrije Universiteit Amsterdam, Amsterdam UMC, Amsterdam Netherlands; ^2^ Alzheimer Center Amsterdam, Amsterdam UMC, Amsterdam Netherlands; ^3^ Fleni, Buenos Aires Argentina; ^4^ Alzheimer Center Amsterdam, Neurology, Vrije Universiteit Amsterdam, Amsterdam UMC location VUmc, Amsterdam Netherlands; ^5^ Alzheimer Center Amsterdam, Department of Neurology, Vrije Universiteit Amsterdam, Amsterdam UMC location VUmc, Amsterdam Netherlands; ^6^ University College London, London United Kingdom; ^7^ Department of Radiology and Nuclear Medicine, Vrije Universiteit Amsterdam, Amsterdam University Medical Center, location VUmc, Amsterdam Netherlands; ^8^ Department of Epidemiology and Biostatistics, Vrije Universiteit Amsterdam, Amsterdam UMC, Amsterdam Netherlands; ^9^ Clinical Memory Research Unit, Lund University, Lund Sweden

## Abstract

**Background:**

Cognitive resilience, the ability to maintain cognitive function despite atrophy, is an important area of research in Alzheimer Disease (AD) and other neurodegenerative disorders. Life‐style factors such as participation in cognitively stimulating activity and physical activity are hypothesized to foster neural connections and enhance reserve, consequently sustaining resilience against cognitive loss. Here, we characterize cognitive and physical activity levels in a large memory clinic cohort with multiple etiologies. Further, we investigate the role of these two factors in resilience to brain atrophy across the AD continuum.

**Methods:**

We included 4033 individuals from the memory clinic‐based Amsterdam Dementia Cohort with multiple diagnoses (Table 1). Levels of (lifetime, past and current) cognitive activity, and (current) physical activity were assessed through self‐reported questionnaires (Cognitive activity Questionnaire, CAQ and Physical Activity Scale for the Elderly, PASE). First, we tested differences in CAQ/PASE scores across the different diagnostic groups in the overall sample using linear models adjusted for age and sex. Second, zooming in on the AD continuum (i.e. amyloid‐β‐positive CU, MCI, or AD), we used linear mixed effects models adjusted for age and sex to assess whether CAQ/PASE had an interactive or additive effect on concurrent cognition and rate of decline (MMSE, memory and executive functioning domains) next to temporoparietal brain atrophy.

**Results:**

Overall, there were differences in lifetime CAQ, current CAQ and current PASE between groups, but not in past CAQ (Figure 1). Within the AD continuum, we found that higher CAQ and higher PASE scores were associated with better cognition at baseline (Table 2). We did not find an interactive effect with brain atrophy on decline, nor an additive effect on the slope in cognition (i.e. higher activity was not associated with reduced speed of decline at similar atrophy levels).

**Conclusion:**

Cross‐sectional effects in the AD continuum are suggestive of a reserve effect in maintaining cognition in disease. While associations with current CAQ and PASE might be (partially) explained by reverse causality, the observed effects with past CAQ suggest that early and mid‐life participation in cognitively stimulating activities provide a cognitive benefit in AD.